# “I am not the same as before”: a mixed-methods study on depression in people with spinal injury in Qatar

**DOI:** 10.3389/fpsyt.2024.1288772

**Published:** 2024-02-21

**Authors:** Badriya Khalifa Al Shamari, Lily O’Hara

**Affiliations:** ^1^ Hamad Medical Corporation, Doha, Qatar; ^2^ Department of Public Health, College of Health Sciences, QU Health, Qatar University, Doha, Qatar

**Keywords:** spinal injury, depression, social support, mixed methods, Qatar, Middle East, Arab

## Abstract

Incidence of spinal injury is high in the Middle East and North African region (MENA) due to the high incidence of road traffic crashes. A spinal injury may trigger mental health issues. Compared to the general population, people with spinal injury are at higher risk for developing major depression, anxiety, post-traumatic stress disorders, substance abuse, and suicide.

**Objectives:**

The objectives of the study were to determine depression prevalence; identify relationships between depression and cause and site of spinal injury, sociodemographic factors, and social support; and explore the lived experiences of depression in people with spinal injury in Qatar.

**Methods:**

A sequential cross-sectional mixed methods study was conducted. In the quantitative component, the universal sample consisted of 106 consenting individuals presenting with spinal injury at Hamad General Hospital, Doha, Qatar between January and December 2020. The Patient Health Questionnaire-9 was used to assess levels of depression and the Medical Outcomes Study Social Support Survey was used to assess perceived social support. The cause and site of injury were obtained from patient records. In the qualitative component, semi-structured in-depth interviews were conducted with 12 purposively selected participants from the quantitative component.

**Results:**

Spinal injury had a negative impact on participants physical, mental, social, and spiritual wellbeing. In total, 69% of participants had some level of depression: 28% mild, 25.5% minimal, and 15% moderate to severe. Depression was not associated with socio-demographic factors, or the cause or site of spinal injury. Higher levels of emotional/informational support and positive social interaction were associated with milder depression. Social support and religious faith were critical in assisting participants to cope with their new situation.

**Conclusions:**

Depression is prevalent among people with spinal injury attending health services. Early detection, referral, and treatment of depression are recommended. Strategies to enhance emotional/informational support and positive social interaction should be developed and tested with people with spinal injury.

## 1 Introduction

Spinal injury is a life-threatening traumatic event associated with significant health burden. The spinal vertebrae typically provide protection to the spinal cords soft tissues; however, they can be displaced causing harmful pressure on the spinal cord resulting in injury. Spinal injury refers to the damage done to either the spinal cord or the vertebrae, ligaments, and disks of the spinal column. The annual global incidence of spinal injury is approximately 40 to 80 cases per million population, with traumatic causes such as motor vehicle crashes, falls, violent acts, and sports-related injuries responsible for up to 90% of these cases (1). The incidence of spinal injury is higher in the Middle East and North African region (MENA) because of the high incidence of road traffic crashes. The proportion of traumatic spinal injuries due to road traffic crashes is 85% in Saudi Arabia (2) and 72% in Qatar (3), the highest rates in the world.

Spinal injury can cause severe physical disability, such as loss of motor, sensory, and other neurological functions (4). In addition, depending on the type of spinal cord injury, complications may include bowel dysfunction, impaired bladder control, sexual dysfunction, mobility restrictions and elevated risk of pressure ulcers (5). A spinal injury may significantly impact a persons daily routine, productivity, and quality of life (QoL) (6) and trigger mental health issues. Compared to the general population, individuals with spinal injury are at higher risk for developing major depression, anxiety, post-traumatic stress disorders, substance abuse, and suicide (7). In turn, depression has a significant negative influence on health and performance of daily living activities after spinal injury (7). Chronic pain due to spinal injury is also associated with depressive symptoms (8). Depression symptoms have been studied extensively among people with spinal injury. Depression is the most common psychological issue affecting approximately 30% of people with spinal injury (9–11) and the risk of depression is higher in those with spinal injury compared to those without (9). Up to 40% of people with spinal injury are clinically depressed during their initial rehabilitation (12, 13). Spinal injury and depression are correlated with longer stays at hospital, prolonged rehabilitation process, less functional improvements and independence as well as lower mobility at discharge (14, 15). If not addressed, depression may lead to negative outcomes such as less independence, more complications, reduced community and social integration, and lower self-appraised health. Depression can also influence the individuals ability to cope with the physiological changes that occur after spinal injury and reduce their motivation to engage in rehabilitation (16). Early detection of depression in people with spinal injury improves quality of life and reduces associated comorbidities (17, 18).

Most research on spinal injury has been conducted in high income countries, with a small number of studies in the Middle Eastern region including Jordan (19), Turkey (20), Kuwait (21), Saudi Arabia (22), Iran (23), and United Arab Emirates (24). However, few studies assess the extent and/or experience of depression in people with spinal injury within the Arab Gulf region, and there have been no studies in Qatar. Such studies are necessary for developing strategies to reduce the risk of depression in people with spinal injury.

## 2 Methods

### 2.1 Study design and population

This cross-sectional mixed methods study aimed to determine the prevalence of depression in people with spinal injury; identify the association between the level of depression and cause and site of spinal injury, sociodemographic factors, and social support; and explore the experiences of depression in people with spinal injury attending health services in Doha, Qatar. The qualitative and quantitative methods were incorporated in all design aspects, conducted sequentially, and had equal value to the study (25). We used the Strengthening the Reporting of Observational Studies in Epidemiology (STROBE) guidelines to comprehensively report the quantitative component of the study and its findings (26).

The study population and sample population for the quantitative component of the study consisted of all individuals attending Hamad General Hospital injury related services including Trauma and Neurological Inpatient Units, Trauma Intensive Care Unit, Trauma Stepdown, Trauma Outpatients Department, and Qatar Rehabilitation Institute Inpatient Unit and Outpatient Department between January and December 2020. Hamad General Hospital is the major governmental healthcare institution in the State of Qatar, where most residents attend in case of emergency, hence it was selected for this study. The use of a universal or census sample enabled us to ensure that there was no selection bias, and that the diverse perspectives of all people with spinal injuries at that time would be included in the study. Eligible participants were: i) males and females; (ii) inpatient and outpatient; iii) aged 18-65 years; iv) recently acquired a spinal injury (spinal cord and spinal column injuries; both traumatic and non-traumatic); and v) conscious and able to communicate. Individuals unable to give informed consent, in a confused state or critical condition, with a history of depression or suicidal attempts, or injured because of a suicide attempt were excluded from the study. A total of 136 individuals met the inclusion criteria; 30 did not consent to participate, resulting in a final sample for the quantitative component of 106 participants. Purposive sampling was used to identify 20 individuals who had completed the quantitative component to approach for the qualitative component of the study, with 12 consenting to participate. Participants were selected to optimize the breadth of participant experiences, with consideration of age, sex, severity of injury, language spoken, and nationality. Purposive sampling is commonly used in the selection of participants that are most likely to result in useful insights. This serves the purpose of providing a credible understanding of the topic under study. Reasons for not participating in the quantitative or qualitative component related to individuals not feeling well enough or not interested in talking at the time.

### 2.2 Data collection

Individuals who met the inclusion criteria were approached at least two weeks after their admission to hospital with a spinal injury by BAS, the principal investigator. The information sheet and consent form were provided in English, Arabic, and Hindi, and explained to participants in the same language by BAS (Arabic and English), and a native Hindi speaking nurse. The data collection window closed in mid-January 2021 to accommodate any individuals that had been injured in late December 2020.

### 2.3 Study measures

#### 2.3.1 Quantitative component

The quantitative component of the study consisted of an interviewer-administered questionnaire-based survey. All questionnaires were administered by BAS in Arabic or English. A native Hindi speaking nurse provided real time translation into and from English with participants who spoke Hindi. Having one questionnaire administrator and one translator reduced the potential for information bias arising from inter-interviewer or translator variability. The questionnaire included socio-demographic items for age, sex, nationality, education, marital status, and number of children. The questionnaire included two existing instruments for assessing depression and social support. Data on the cause and site of the injury were collected from participants medical records.

##### 2.3.1.1 Patient health questionnaire

As there is no spinal injury-specific instrument for measuring depression, the Patient Health Questionnaire (PHQ-9) (27) was used to assess the severity of depression, consistent with other studies on the psychological effects of spinal cord injury (28). This questionnaire is the only instrument that aligns with the Diagnostic and Statistical Manual of Mental Disorders criterion that clinical diagnoses should be based on participants experiences in the past two weeks. PHQ-9 consists of nine items each rated from 0 (not occurring at all) to 3 (occurring nearly every day) with a total score ranging from 0 to 27. Results are categorized as minimal (1–4), mild (5–9), moderate (10–14), moderately severe (15–19), and severe depression (20–27). The English, Arabic, and Hindi versions of PHQ-9 were used in this study. The English version of the PHQ-9 version has been used extensively in people with spinal injury (11, 29, 30) and has high internal reliability (18). Internal reliability of PHQ-9 in this study was also high (Cronbachs α = .812.) The Arabic version of the PHQ-9 has been validated in a sample of individuals in Lebanon. The instrument had high internal reliability (Cronbachs α = .823) and reliability (31). No studies have validated the Hindi version of PHQ-9.

##### 2.3.1.2 Medical outcomes study social support survey

The Medical Outcomes Study Social Support Survey (MOS-SSS) (32) identifies self-reported information regarding companionship, assistance, and other types of support including emotional and affectionate support. This instrument was originally created for individuals with chronic conditions; positive associations were observed between measures of social support and psychological health (32). The MOS-SSS is a 19-item questionnaire that yields an overall social support index and four subscale scores for emotional/informational support, tangible support, affectionate support, and positive social interaction. Emotional/informational support refers to perceived availability of someone to talk to about personal issues, the availability of advice or information when needed, and the expression of love and affection. Tangible support refers to the availability of practical assistance or material aid. It assesses the perceived availability of help with daily chores, financial assistance, or other forms of tangible support. Affectionate support refers to the perceived availability of love, affection, and expressions of care. It reflects the emotional aspects of support, emphasizing the importance of warmth and closeness in relationships. Positive social interaction refers to the perceived availability of someone to have fun with, to relax and enjoy time with, and to engage in recreational activities or social events. It focuses on the positive aspects of social interaction and companionship. Participants were asked to select the frequency of situations within each of these four domains of social support. Response scores are based on a five-point scale ranging from 1 (none of the time) to 5 (all the time). An overall social support index is determined by calculating average responses of the 19 items, yielding a score ranging from 15. Subscale scores are determined by calculating average responses for subscale items, yielding scores ranging from 15 for each subscale. Higher subscale and overall scores indicate more social support. The English version of the MOS-SSS has been validated in numerous studies and found to have high internal validity and reliability (32, 33). The Arabic version of the MOS-SSS has been validated and found to have high validity and reliability (34). A psychometric review of various translations of MOS-SSS recommended that the Arabic version has potential use in future research and practice (33). In this study, the MOS-SSS had excellent internal reliability (Cronbachs α=.960), as did the subscales: emotional/informational support (α=.955), tangible support (α=.950), affectionate support (α=.922), and positive social interaction (α=.967). No studies have validated a Hindi version of the MOS-SSS.

#### 2.3.2 Qualitative component

##### 2.3.2.1 Semi-structured interview

A semi-structured interview was conducted to collect in-depth qualitative data on participants experiences with depression. Interviews were conducted by BAS in Arabic or English. A native Hindi speaking nurse provided real time translation into and from English with participants who spoke Hindi. As with the interviewer-administered questionnaire, having one interviewer and one translator reduced the potential for information bias arising from inter-interviewer or translator variability. Interview questions focused on participants feelings related to their injury over the past two weeks, how their injury contributed to such feelings, and how they were dealing with the consequences of their injury. Given the extent of participants medical condition, interviews were kept relatively short, lasting an average of 15 minutes. Interviews continued until data saturation was reached and no new information was emerging (35).

### 2.4 Data analysis

The mixed methods design included concurrent data analysis whereby quantitative and qualitative data analyses were completed separately, and results from analyses were then compared (36). Quantitative data analyses were completed using statistical software package IBM SPSS Statistics version 25.0 (SPSS Inc., Chicago, IL, USA). Initially, data were screened for outliers or data errors. There were no missing data due to the questionnaire being interviewer-administered. Descriptive statistics were calculated for all measures; continuous data are presented as mean ± Standard Deviation (SD), and categorical data are summarized as count (percentages). The proportion of participants in each depression category was calculated. A binary variable was then created by grouping mild, moderate, moderately severe, and severe categories of depression (labeled ‘depression). Pearsons χ^2^ test was used to test the association between depression and categorical independent variables. Pearson correlation coefficients (r) were calculated to evaluate the association between the continuous PHQ-9 score and MOS_SSS scores. The level of statistical significance was set at P<0.05.

Thematic analysis was used to analyze the interview data via the four-step method of data preparation, data reduction, displaying data, and verifying data (37). Data preparation involved verbatim transcription of interviews in English and Arabic. Arabic transcriptions were translated into English. Interviews in Hindi were transcribed in English after real time translation. Data reduction included line by line coding, looking for similar concepts, categorizing concepts, and grouping them into larger themes. Data are displayed in the results section according to themes and categories, supported by direct quotations from participants. Quotes are short due to the medically required brevity of the interviews. Data verification was ensured by cross-checking results with the original transcripts (38). All data analysis took place in 2021.

### 2.5 Ethical considerations

The study was conducted in full compliance with the Declaration of Helsinki, Good Clinical Practice (GCP), and within the Ministry of Public Health laws and regulations in Qatar. Ethical approval was obtained from Medical Research Center of Hamad Medical Corporation and Qatar Universitys Institutional Review Board (QU-IRB 1341-E/20). To preserve the anonymity of participants, the specific nationality of participants is not reported, and instead, nationalities are grouped into regions. Pseudonyms are used for quotations when reporting qualitative data.

## 3 Results

### 3.1 Quantitative results

A total of 106 participants were included in the study. The average age was 36 years, and the majority were male, from Asia, married, had three or fewer children, and some level of education (**Table 1**). The two main causes of injury were motor vehicle crashes (38%) and falls (37%). Fewer injuries were caused by pedestrian accidents (10%), work-related accidents (9%), and back pain (4%). Most injuries affected the lumber spine (62%), followed by thoracic (39%), cervical (36%), and sacral spine (10%). Motor vehicle crashes were the most common cause of injury that resulted in thoracic neurological deficit (46.2%) and half of sacral spine injuries were caused by falls.

**Table 1:** Demographic characteristics of study participants (N=106).

	Mean ± SD or N (%)
**Age** (years)	35.82 ± 10.00
**≤35**	59 (55.7)
**>35**	47 (44.3)
Sex	
Male	100 (94.3%)
Female	6 (5.7%)
Marital status	
Married	72 (67.9)
Single	34 (32.1)
Total number of children	
0	39 (36.8)
1-3	50 (47.2)
4-6	11 (10.4)
7-9	6 (5.7)
Nationality region	
Asia	72 (67.9)
Africa & Europe	10 (9.4)
Middle East	24 (22.6)
Education	
Uneducated	20 (18.9)
School	61 (57.5)
University	25 (23.6)

The mean PHQ-9 score was 4.82 ± 5.25 indicating mild depression. Overall, 31% of participants had no depression and 69% had some level of depression: 28% mild, 25.5% minimal, 7% moderate, 7% moderately severe, and 0.9% severe depression. There were no statistically significant relationships between depression and the cause of injury, site of injury, age, sex, nationality, education, marital status, or number of children (**Table 2**).

**Table 2:** Association between depression and cause of injury, site of injury, and sociodemographic factors (N=106).

	Depression n=73 (69%)	No depression n=33 (31%)	P value
	n (%)	n (%)	
**Cause of injury**			0.65
Fall	28 (71.8)	11 (33.3)	
Motor Vehicle	25 (62.5)	15 (37.5)	
Pedestrian	9 (81.8)	2 (18.2)	
Work Related	7 (9.6)	3 (9.1)	
Back Pain	2 (50)	2 (50)	
Other	2 (100)	0 (0)	
**Site of injury**			0.92
Cervical spine	25 (69)	11 (31)	
Thoracic spine	25 (64)	14 (36)	
Lumber spine	44 (71)	18 (29)	
Sacral spine	7 (70)	3 (30)	
**Age**			0.27
≤35	38 (64)	21 (36)	
>35	35 (75)	12 (25)	
**Sex**			0.09
Male	67 (67)	33 (33)	
Female	6 (100)	0	
**Nationality**			0.20
Asia	46 (64)	26 (36)	
Africa & Europe	7 (70)	3 (30)	
Middle East	20 (83)	4 (17)	
**Education**			0.99
School	42 (69)	19 (31)	
University	17 (68)	8 (32)	
Uneducated	14 (70)	6 (30)	
**Marital status**			0.79
Married	49 (68)	23 (32)	
Single	24 (71)	10 (29)	
**Number of children**			0.095
0	28 (72)	11 (28)	
1-3	33 (66)	17 (34)	
4-6	10 (91)	1 (9)	
7-9	2 (33)	4 (67)	

The overall mean MOS-SSS score was 4.12 ± 0.99. The subscale scores were affectionate support 4.25 ± 1.19, emotional/informational support 4.23 ± 1.03, positive social interaction 4.04 ± 1.26, and tangible support 3.9 ± 1.4. There was no significant association between PHQ-9 score and the overall social support index (r = -0.189; P = 0.053) (**Table 3**). Significant but weak inverse correlations were observed between PHQ-9 score and emotional/informational support (r = -0.202; P < 0.001), and positive social interaction (r = -0.210; P < 0.001). Participants with higher emotional/informational and positive social interaction had lower levels of depression.

**Table 3:** Association between Patient Health Questionnaire-9 (PHQ-9) score and Medical Outcomes Study Social Support Survey (MOS-SSS) scores (N=106).

	R	P value
Overall Social Support Index	-0.189	0.053
Emotional/Informational support	-0.202	0.001*
Tangible support	0.045	0.648
Affectionate support	-0.120	0.221
Positive social interaction	-0.210	0.001*

*Correlation significant at the 0.05 level (2-tailed).

### 3.2 Qualitative results

**Figure 1:**
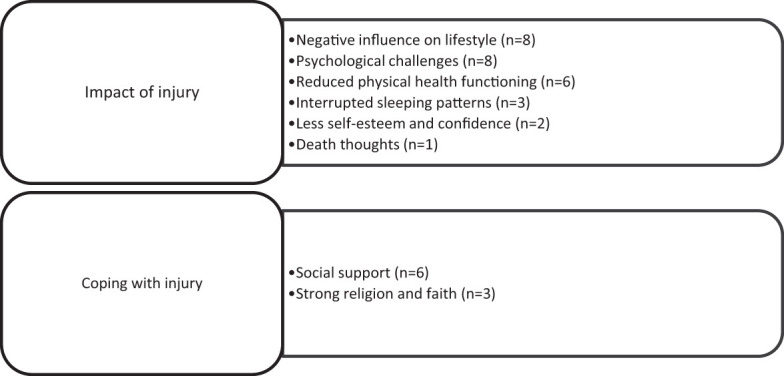
Themes, subthemes, and number of participants reporting each sub-theme.

A total of 12 participants aged 20 to 46 years with eight different nationalities participated in the interviews. Nine participants had motor vehicle-related injuries, two had injuries caused by falls, and one had a pedestrian injury. Two thematic categories emerged relating to the impact of the spinal injury and coping with the injury. Themes related to the impact of injury were negative influence on lifestyle, less self-esteem and confidence, psychological challenges, reduced physical health functioning, interrupted sleeping patterns, and death thoughts. Themes on coping with injury were strong religion and faith, and social support (**Figure 1**).

#### 3.2.1 Thematic category 1: Impact of the injury

##### 3.2.1.1 Negative influence on lifestyle

Most participants struggled with their daily routines after injury, noting they were not the same person as prior to injury. One participant Zebiba mentioned she was unemployed after the injury, saying, “I stopped working for the time being. I dont even do anything at home, my husband and sister help me. My daughters stopped going to school because I am unemployed and going through financial problems.” Yasser also noted the significant effect on him, saying “Injury had a great impact on my life, but I try not to think much about it.” Another participant reported not being able to take care of her children. A few said that they are still fighting hard and struggling to overcome their situation. They mentioned that due to the pain, they are less active after the injury. Sara explained, “Before the accident, I was very active but now I can barely move because of the pain. I am not the same as before; I cant move and get out or take care of my children. These things affect me psychologically … Until now, I am still not allowed to carry my kids or any heavy things. I am even not allowed to move a lot.” Muhanad said, “I try to fight this, and I try to look for ways to overcome my situation.”

##### 3.2.1.2 Psychological challenges

Most participants reported feeling sad and losing interest in everything. Some participants felt useless and helpless. Some participants also reported guilty feelings about the impact on their families due to their lack of ability to perform tasks independently. As Randa explained, “I sometimes feel sad and useless … not depressed, and not all times … I dont see myself as before … sometimes I feel that I lost interest in everything.” Sara expressed similar ideas, saying, “I feel that I am useless; I was strong before but now I feel like I need people to help me. I cannot be independent like I was before.” One participant stated that pain caused distress as well as mood swings which affected the performance of certain activities. Another reported that being away from family was the main cause of emotional and psychological stress. Muhanad explained, “A person feels distressed when not being able to move and perform family and social obligations Mood changes when in pain. If I need to do something but cannot … it affects my mental health.” In contrast to these expressions of ongoing psychological distress, two participants reported being depressed only at injury time and then feeling normal with time. One participant mentioned that performing physical activity had a positive influence and improved their psychological wellbeing.

##### 3.2.1.3 Reduced physical health functioning

Most participants reported difficulty in movement, pain, and tiredness, lacking energy and feeling weak. A few participants lost their appetite. Zenon said, “I cant walk, and I cant do any work now because of this back pain. … Even when someone is helping me, I cant walk.” Jassim noted that “I cannot run like before.”

##### 3.2.1.4 Interrupted sleeping patterns

Some participants struggled with insomnia due to overthinking and distress or trouble breathing while sleeping due to pain. They were not getting enough sleep, and this impacted their recovery.

##### 3.2.1.5 Reduced self-esteem and confidence

Some participants reported having lower self-esteem and confidence due to the trauma caused by the incident. Randa explained the situations in which she felt most affected, saying, “I feel afraid of going down the stairs and while going out of the house, even if I see a car; I am suffering from a trauma in my daily activities” and Zebiba also noted, “I feel afraid whenever I see a car.”

##### 3.2.1.6 Death thoughts

One participant, Zebiba, reported having suicidal thoughts due to the unbearable pain. Death was perceived as a way to relieve sorrow and pain. She said, “In the beginning, I was thinking of death and saying Oh God relieve me. I was in so much pain, I just wanted to end everything.”

#### 3.2.2 Thematic category 2: Coping with the injury

##### 3.2.2.1 Social support

Half the participants stated that social support, particularly from family, was the most important factor for coping with the injury. Family gatherings, quality time with their spouse and children, outings, and visits from friends were all found to ease the impact of the injury. They felt stronger because of the care offered by family members and friends. As Ibrahim said, “Everyone cares for me now … I feel a lot of happiness when a friend visits me.” Hamad shared, “I got married after the injury and this is what helped me overcome my injury condition.” Randa explained the impact of her familys support, saying “When I talk to my family, I feel much better, and I forget my sorrow. But when I am alone in my room or when going out alone, I feel sadder.” One participant felt that social support from the medical staff was important, saying that conversations with the doctors provided relief.

##### 3.2.2.2 Strong religion and faith

Three participants mentioned that religion and faith were the main factors for coping with their injuries. They reported that their belief and faith in God almighty were strong and ultimately helped them to cope with their situation. As Sara said, “I feel I am closer to God; I have a bigger faith and thankful I am alive … I accept my destiny and I have hope that I will recover.” Muhanad said, “My strong belief in God almighty makes me more hopeful and accepting.”

## 4 Discussion

This cross-sectional mixed methods study aimed to determine the prevalence of depression in people with spinal injury; identify the association between the level of depression and cause and site of spinal injury, sociodemographic factors, and social support; and explore the experiences of depression in individuals with spinal injury attending injury-related health services in Qatar. The questionnaire-based survey found that the prevalence of depression among the participants was high, with 69% of participants having some level of depression, including 15% with moderate to severe depression. Depression was not associated with socio-demographic factors, or cause or site of spinal injury. Interviews confirmed that spinal injury had a negative impact on participants lives, including their physical and mental health, as well as social and spiritual wellbeing. Participants struggled with their daily routines after the injury and had difficulties in their day-to-day activities, which had an impact on their self-worth and self-esteem. Some participants reported significant psychological challenges, including suicidal ideation. The highest levels of social support were affectionate support and emotional/informational support. Emotional/informational support and positive social interaction were inversely correlated with depression. This was consistent with the findings of the interviews, which highlighted the important role of social support in improving participants ability to cope with their new situation. Social support, specifically family support, and participants spiritual support and religious faith were critical in assisting them to cope with their new situation.

Our findings on the high prevalence of depression in people with spinal injury are consistent with many other studies. A study across six countries found that for people with a physical injury, the odds of depression were 72% higher than people without injury, and the odds for those with an injury with a disability were 3.81 times higher than for those without injury (39). Another study found that for those hospitalized with traumatic injury, the severity of the injury was associated with the development of depression (40). Given that injury to the spine is likely to be severe and result in disability, the site of the injury is an important factor in the development of depression. Other research confirms that individuals with spinal injury are at higher risk for developing major depression, anxiety, and post-traumatic stress (41). The prevalence of depression in our study was slightly lower than in a study in Iraq, which found that 86% of study participants were depressed (42). Similar to other studies, this study found no significant associations between depression and sociodemographic factors (14, 18, 43, 44), which indicates that depression is not selective with respect to sociodemographic characteristics; it appears everyone is equally vulnerable. In contrast, other studies have found relationships between depression and sociodemographic factors, whereby females, less educated, and older age groups had a higher risk for depression (45). Such inconsistencies in results imply that the association between sociodemographic factors and depression is still not clearly identified and requires further investigation.

The finding from this study that spinal injury had a negative impact on participants daily routine, physical, mental, social and spiritual health is consistent with other studies in which people with spinal injury experienced confusion, tiredness, pain, stress, low self-efficacy, inadequate sleep, and reduced involvement in day-to-day activities and social integration (9, 46–48). Chronic pain conditions can increase suicide among people with spinal injury (49) with more than 50% of people with spinal injury having suicidal thoughts (49, 50). Although only one participant in this study had suicidal ideation in the early stages of injury, participants may have not been fully honest due to fear and the fact that suicide is forbidden from a religious perspective (51) or stigmatized from a cultural perspective (52); thus, it may have been underreported. In some religious and cultural communities, mental health issues, including suicidal thoughts, are highly stigmatized. Individuals may fear judgment, social exclusion, or shame, leading to underreporting or reluctance to seek help. Such stigma has been demonstrated in Asian (53) and Arab countries (54), the two largest groups of participants in our study. Cultural norms also play a role in shaping how emotions and mental health are expressed. Some cultures emphasize emotional restraint or discourage open discussion of personal struggles, which may hinder people from openly sharing their mental health concerns (52), including thoughts of suicide. Asian and Arab cultures emphasize collectivism and prioritizing the wellbeing of the family or community over individual concerns, which may have led to participants being less inclined to disclose personal struggles, including mental health issues, to avoid burdening their loved ones.

Both quantitative and qualitative results demonstrated the importance of social support in coping with spinal injury. Likewise, the role of social support in coping with spinal injury has been highlighted in other studies (49, 55, 56). Social support is a crucial factor for reducing depression severity, improving coping ability, and injury recovery (57). In our study, emotional support, particularly from family was critical, and this is consistent with other studies (58). When family support is not available, peer support is effective (58). Given the large expatriate community in countries such as Qatar and the Gulf Cooperative Council (GCC), with many people not having family living with them, it is important for health institutions to facilitate peer support programs for people with spinal injury.

In this study, spiritual support played a significant role in helping participants to cope with their situation. Participants felt that having strong spiritual beliefs provided them with the strength required to address the needs arising from their new situation. This was consistent with other studies that also highlight the important role of spirituality in coping with spinal injury (56, 59, 60).One of the first studies conducted in a primarily Muslim country found that spiritual wellbeing was an important factor contributing to the capacity of individuals to cope with the physical, social, economic, and emotional issues arising from the injury (61).

Although we found no statistical association between depression and the cause or site of the spinal injury, we did not test whether these factors played a role in the relationship between depression and social support. Future studies should examine the potential mediating role of the nature of the spinal injury, as this may contribute to the type and intensity of social support required. The level and severity of the injury and the degree of physical independence will impact the level of practical and financial support required for mobility, daily activities, self-care, rehabilitation, and medical needs, as well as the degree of emotional support required (62).

This study had several strengths and limitations that must be considered in interpreting the results. A significant strength was the census sample whereby all individuals attending Hamad General Hospital injury-related services over one year were included. This enhances the generalizability of the results. The risk of variation in data collection was reduced by the principal investigator administering the questionnaire and conducting the interviews. The availability of instruments and interviews in three languages helped to optimize the number of participants from different nationalities, however Hindi versions of the PHQ-9 and MOS-SSS have not been validated, reducing the trustworthiness of results for participants speaking Hindi. Data collection was undertaken for a minimum of two weeks from injury onset, which may have impacted participants self-assessment of their mental state. The study did not determine how levels of depression may change over time. Most participants were migrant workers and may have feared deportation which might have caused underreporting. Conducting subgroup analyses with relatively small samples in this study may have resulted in type 2 errors. The methodological limitations and the specific context of Qatar with its high prevalence of migrant workers limit the generalizability of the findings to other countries.

This study provides a novel and important addition to research in Qatar and the region. Depression is prevalent among people with spinal injury and is associated with lower levels of social and spiritual support, but not sociodemographic factors or injury cause or site. Many of the poor outcomes associated with spinal injury arise from insufficient medical care and rehabilitation services, coupled with obstacles in physical, social, and policy environments (63). In addition to the direct impact of spinal injury on affective disorders, policy decisions that impact on care and support for people with spinal injury may also adversely affect neurological recovery, resulting in further increased levels of depression as well as other personal and economic burdens for both individuals and the health system (63). Delivering comprehensive care that includes depression diagnosis and treatment during acute and post-acute phases can mitigate the likelihood of depression and enhance overall quality of life by maximizing functional capabilities, promoting independence, ensuring overall wellbeing, and facilitating community integration to enable people with spinal injury to live full and meaningful lives (64). At a minimum, spinal injury care programs should include rehabilitation psychologists to facilitate the early detection of depression in people with spinal injury and timely referral to treatment (64). Beyond this, access to valid, reliable, and consistent data is required for policy makers to develop evidence-based actions, and a spinal injury registry could provide access to data to support evidence-based practice to optimize continuity of care, rehabilitation services, and social support for people with spinal injury (63).

All research creates the need for more research, and this study is no different. Knowing that people with spinal injury are at higher risk for depression is important. More research is now required on the impact of policies that enable culturally safe interprofessional collaborative practices for the early detection, referral, and treatment of depression in people with spinal injuries. Research is also required on the impact of programs developed by health services or within the social sector that provide more structured peer support for people with spinal injuries.

## Data availability statement

The raw data supporting the conclusions of this article will be made available by the authors, without undue reservation.

## Ethics statement

The studies involving humans were approved by Qatar University Institutional Review Board. The studies were conducted in accordance with the local legislation and institutional requirements. The participants provided their written informed consent to participate in this study.

## Author contributions

BS: Conceptualization, Data curation, Formal analysis, Funding acquisition, Methodology, Project administration, Writing – original draft, Writing – review & editing. LOH: Supervision, Methodology, Writing – review & editing.
